# User Experience of Mobile Personal Health Records for the Emergency Department: Mixed Methods Study

**DOI:** 10.2196/24326

**Published:** 2020-12-15

**Authors:** Su Min Kim, Taerim Kim, Won Chul Cha, Jae-Ho Lee, In Ho Kwon, Yuri Choi, June-Sung Kim

**Affiliations:** 1 Department of Digital Health Samsung Advanced Institute for Health Science & Technology Sungkyunkwan University Seoul Republic of Korea; 2 Department of Emergency Medicine Samsung Medical Center Sungkyunkwan University School of Medicine Seoul Republic of Korea; 3 Health Information and Strategy Center Samsung Medical Center Seoul Republic of Korea; 4 Department of Emergency Medicine Asan Medical Center University of Ulsan College of Medicine Seoul Republic of Korea; 5 Department of Information Medicine Asan Medical Center University of Ulsan College of Medicine Seoul Republic of Korea; 6 Department of Emergency Medicine Dong-a University Hospital Busan Republic of Korea; 7 Department of Emergency Medicine Dong-a University College of Medicine Busan Republic of Korea

**Keywords:** personal health records, mobile health, patient engagement

## Abstract

**Background:**

Personal health records (PHRs) can be useful in the emergency department, as they provide patient information in an accurate and timely manner and enable it to be used actively. This has an effect on patients’ health outcomes and patient experience. Despite the importance of PHRs in emergencies, there are only a few studies related to PHRs in emergencies that evaluate patient experience.

**Objective:**

This study aims to introduce the novel mobile PHR (mPHR) platform to emergency environments and assess user experience.

**Methods:**

The study was conducted from October 2019 to November 2019. In total, 1000 patients or carers in the emergency departments of 3 hospitals were provided an application-based service called FirstER, which was developed to collect and utilize medical information for patients in the emergency department. This study was performed as a mixed methods study. After using FirstER, we investigated its usability and conducted a survey on the experience of obtaining medical information with a legacy system and with FirstER. Additionally, we interviewed 24 patients to gain insight into their experiences regarding medical information using FirstER. For the quantitative analysis, the survey results were analyzed using descriptive statistics (mean and standard deviation). For the qualitative analysis, we determined the keywords and their frequencies from each survey question and interview question.

**Results:**

In total, 1000 participants, consisting of both patients and carers, were recruited in this study. Their mean age was 41.4 (SD 13.3) years. We ascertained participants’ satisfaction with FirstER and their mPHR needs through a survey and an in-depth interview. With the current system, participants were not well aware of their health conditions and medical information, and they were passive in the use of their medical information and treatment. However, they wanted their medical information for several reasons, such as information sharing and managing their health conditions. FirstER provided participants with their needed information and an easy way to access it. The mean System Usability Scale (SUS) value was 67.1 (SD 13.8), which was considered very near to acceptable.

**Conclusions:**

This study is the first to implement mPHRs in the emergency department of large tertiary hospitals in the Republic of Korea. FirstER was found to enhance user experience in emergencies, as it provided necessary medical information and proper user experience. Moreover, the average SUS was 67.1, which means that participants found FirstER to be very near to acceptable. This is very encouraging in that FirstER was developed within a very short time, and it was a pilot study.

**Trial Registration:**

Clinicaltrials.gov NCT04180618; https://clinicaltrials.gov/ct2/show/NCT04180618

## Introduction

### Importance of Information in the Emergency Department

Patient information is very important in the emergency department. Medical staff can treat patients appropriately when they have accurate patient information in a limited time, which affects the patients’ health outcome [1-4]. From the patient's point of view, some studies show that a more positive experience was had in terms of improved health outcomes or emotional aspects during the treatment process when the patient received medical information about themselves [5-8]. However, it is relatively difficult to obtain or provide medical information for emergency patients as compared to other patients, such as outpatients; handling patient information is challenging because they visit the hospital unexpectedly and patient information is often managed by a separate hospital [4-6]. Therefore, it is important to develop ways for providing patient medical information to satisfy the special situations and needs of emergency patients, which, ultimately, contribute to the improvement of health outcomes [9].

### Patient Health Records Enhance Care Effectiveness by Providing Patient Medical Information

Despite the importance of access to and provision of complete patient information, access to on-demand medical information is not well achieved [10]. In this situation, a feasible solution is the patient health record (PHR), which allows patients to generate and manage their overall information [11-16], as well as the mobile PHR (mPHR), which is linked with mobile phones and can access information from anywhere. These enable continuous care and follow-up by aggregating patient information that had previously been fragmented around medical institutions for the patient's focus [14,17]. In addition, by better understanding one's health through one's own medical information, patients can actively participate in treatment-related activities such as decision-making and medication compliance, which positively affect self-management effectiveness. Consequently, it improves care outcomes and patient status [11-16].

### Importance of User Experience of PHR for the Emergency Department

We expected to improve the patient experience by providing information through mPHR in emergency situations. However, studies conducted thus far have focused on PHR research in nonemergency situations [13,15]. In the context of emergency situations, there were studies on instruction and education to improve the discharge process of patients, but this did not utilize PHR [5]. For PHR in emergency situations, some studies were conducted with respect to setting systems for PHR [18]. Additionally, a study reported that both patients and medical staff were willing to use PHR [18]. Although many studies have suggested that PHRs have a positive effect on patients, it is necessary to observe the user experience of mPHR in real emergency situations because the utility of a system depends on a specific environment [19]. We wanted to ensure that we provided easy-to-understand patient information that was genuine and updated [6,20,21] and that the patients could use the mPHR application well [6,20-22]. Research on these patients’ experiences will lead to better patient participation and satisfaction, and ultimately, may achieve the goal of improving the quality of health care [20,23].

### Objective

This study aimed to introduce the novel mPHR platform to emergency environments and to assess user experience. 

## Methods

### Development of the Novel Mobile PHR: FirstER

FirstER, a mobile application-based platform, was developed to collect and utilize medical information, especially for emergency patients and medical staff. Our previous study described the content of FirstER [24]. We conducted interviews and surveys with various stakeholders in emergencies to identify PHR service requirements in emergency medical environments, services and functions based on PHRs, patients' willingness to provide information for PHR services, and items of medical value for PHR development. That study proved the validity of the need for PHRs in the emergency medical environment and was used as basic data before implementing practical services; therefore, it became the basis for organizing the information used in this study.

Further, medical staff reassessed the results to determine the most necessary details and organized the information. Later, the whole system was created, including a mobile application for patients and a web page for medical staff. Servers were set up in each hospital, and they were connected with a security cloud service so that the medical information from 3 hospitals could be gathered in a cloud assigned for each patient. With the mobile application and web page, patients and medical staff could access the medical information of the patients in the emergency department.

### Structure of the FirstER

#### Overview

The following process was carried out: First, when a patient visited the emergency department, they downloaded the FirstER application on their mobile phone, after which the patient created an account and agreed to provide personal information. Second, the cloud received the subscriber’s information (including patient ID) and sent it to the linked server in the hospital system. Third, the subscriber’s emergency department data were extracted from the hospital information system server, and the subscriber’s data were sent to the service-linked server. Fourth, the service-linked server sent the subscriber’s emergency department data to the cloud. Fifth, in the cloud, the emergency department data were stored and sent to the application. Lastly, the patient could check their emergency department data on their mobile phone.

**Figure 1:**
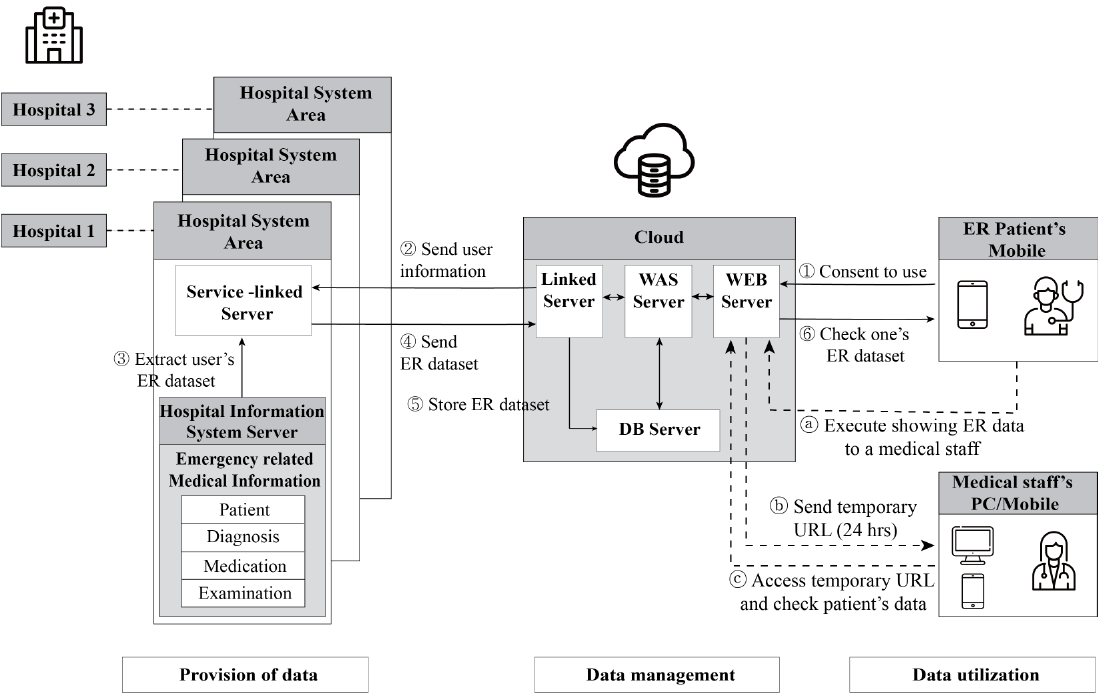
Overview of the FirstER system. ①~⑥ (line): the process in which patients obtained their medical information. ⓐ~ⓒ (dotted line): the process in which medical staff accessed the patient’s medical information. When a user sent a request to the client, the webserver processed the command and sent an answer back to the user. The user requests that the webserver could not process were sent to the WebSphere Application Server (WAS), and the results were handed over to the users after they were received. It provided static content such as HTML, CSS, etc. The WAS server provided dynamic content, such as DB inquiry, processing logic, etc. ER: emergency room; DB: database.

Once the patient agreed to reveal and share their medical information with medical staff through an emailed link to a medical information summary, the medical staff could connect to the web page showing the patient's medical information (Figure 1).

**Figure 2:**
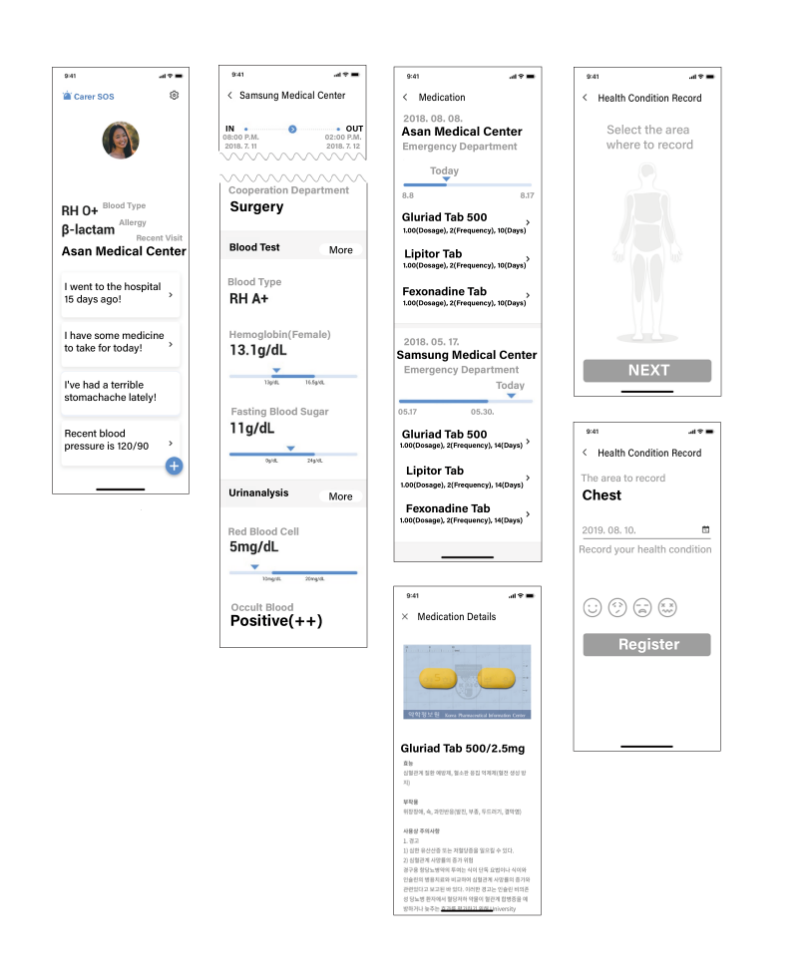
Screens of the FirstER application. (a) The main page shows the user’s brief information for an emergency, such as name, gender, age, blood type, allergy, and recent hospital visits. Additionally, there is a button for sending a message to the user’s carer in case of an emergency. Moreover, there are 4 sections for hospital visit records, medication, pain, and blood pressure. (b) Hospital visit records are presented; when the user clicks on a hospital visit record, the user can access specific information, such as lab test results. (c) Medication records allow the user to see records of prescribed medications. (d) FirstER has self-record components for the user to input their pain and blood pressure; on the Pain record screen, the user can choose where they feel pain and how it felt, and the user can input brief, descriptive text. The app screen language was translated to English.

FirstER consists of 2 categories: health records, and information management and setting (Figure 2).

#### Health Records

The health records comprised 2 sections: hospital records and self-reported information. The hospital records involved visit records and information on medication. When the patient who created an account in the service logged into the application, they could see the main page, which featured a summary of their records (name, gender, age, blood type, and recent hospital visits) at the top of the page. Additionally, there were 4 buttons showing hospital visits, medication, self-reports of pain, and blood pressure. From the main page, the patient could download the emergency department information by entering the patient ID. Under “Hospital Visit Records,” there were details of the reason for the visit, the patient’s status at that time, and multiple lab results, such as blood, urine, and biochemical examination. Under “Medication Information,” users could see the name and in-depth information about the prescribed drug.

In the “Self-report” section, the user could select body regions in an image of the human body to indicate where they feel pain and enter their symptoms with an icon expressing the severity of the pain. Additionally, the user could record their blood pressure. These self-reports could be used in medical visits or for the user’s health self-management.

As the main feature of FirstER, the user could send the medical information summary to their carers and medical staff.

#### Information Management and Setting

The Information Management and Setting page allowed users to manage individual, health, and policy information and access help. Under “Individual Information Management,” the patient could manage their information, including patient ID for each hospital and passwords. In the “Health Information Management” section, the patient could choose which data are visible (in case the patient believed that some information was unnecessary). Additionally, a user guide, contact information for asking questions, and policy information was featured. Moreover, the user could decide to withdraw from the service without any constraint in this section.

### Study Design

#### Participants

The participants recruited for this study had visited the Emergency Department of Samsung Medical Center (SMC), Asan Medical Center (AMC), or Dong-A Medical Center (DMC) from October 2019 to November 2019. These 3 medical institutes are large tertiary hospitals in Seoul and Busan in Korea; the number of beds in each hospital are 1989 in SMC, 2705 in AMC, and 1000 in DMC. A System Usability Scale (SUS) score of 68 was considered an average score, and a score of 70 was the basis for acceptability. Therefore, 69.5 points (ie, slightly above 68) was set as the target SUS value. The method for calculating the number of samples used a constant of (68) and a difference of (69.5-68=1.5). To analyze the mean values, a 1-sample *t* test was chosen. The effect size was 0.117, the significance level was 0.05, and the power was 0.95. Effect size was obtained using the following formula:


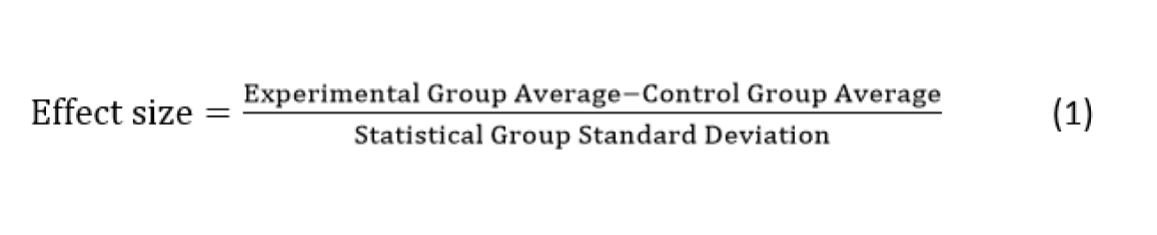


Under these conditions, a required sample size of 792 was calculated and 1000 people were recruited for multicenter research, taking a dropout rate of approximately 21% into account.

**Figure 3:**
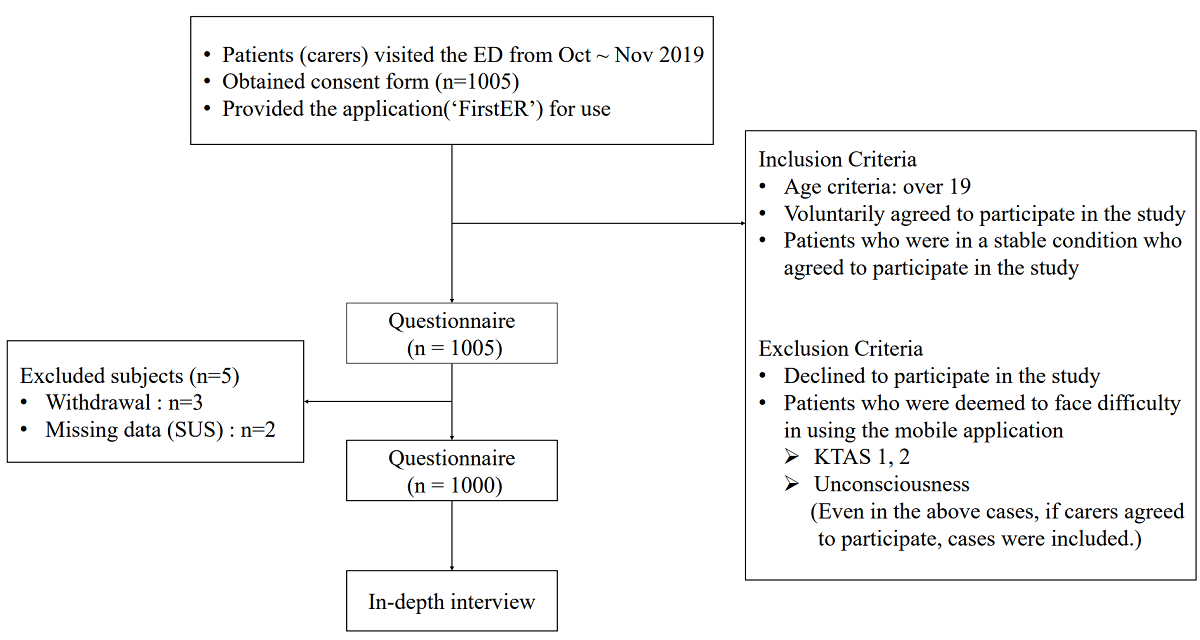
Flowchart for the study, featuring inclusion and exclusion criteria. ED: Emergency Department; KTAS: Korean Triage and Acuity Scale (a Korean emergency patient classification tool divided into levels 1-5 depending on severity); SUS: System Usability Scale.

Participants were excluded if they were younger than 19 years, declined to participate, were deemed to face difficultly using mobile applications, or were unconscious while leaving the emergency department (eg, if the patient was disoriented or confused, not conscious, or whether they were in a state of shock or cardiac arrest). However, even in these cases, carers were able to be included as clinical trial subjects. Informed consent was obtained from all the participants. The study protocol (2019-07-066-010) was approved by the institutional review board (IRB) office at Samsung Medical Center in Seoul, Republic of Korea (Figure 3).

#### Protocol

We conducted a usability study with a fully functional mobile phone prototype using a mixed methods approach. When the patients and carers visited the hospitals’ emergency departments, they were informed of the study's purpose and provided with consent forms. Further, they could download the FirstER application, and they were informed about how to use it. After they installed the application, they were asked to complete a paper questionnaire, including information on demographic characteristics, a smartphone-use score, surveys, and a SUS score. After that, in-depth interviews were conducted with semistructured questions, including details of the survey’s content. The interview was conducted via convenience sampling. All the interviews were recorded with the consent of each participant (Figure 3).

#### Outcome Measurement

The primary outcome was the application's usability as reported by the users (ie, patients and carers) and how they felt while using FirstER. For this, we used the survey method, an in-depth interview, and SUS scores.

#### Questionnaire Study

The questionnaire consisted of 4 categories: (1) demographic information, (2) smartphone-use status, (3) survey on the experience of obtaining medical information with a legacy system and FirstER, and (4) the SUS for assessing the usability of the application using a 5-point Likert scale (1-strongly disagree, 5-strongly agree) [25].

In the demographics category, participants were asked about their age, gender, marriage status, main carer, education level, and residential area. For smartphone-use status, we obtained 10 questions from a smartphone technology quotient and indices for smartphone usage developed in Korea, which consisted of 4 parts: recognition, access, usage, and capacity [26]. The questions were about the period and duration of smartphone use, how important the smartphone and its applications were in the participants’ life, how well the participants could use applications, and so on. A 5-point Likert scale was used to respond to these questions (1-strongly disagree, 5-strongly agree), except for questions 7 and 8, for which a 4-point Likert scale was used. For each question, the responses were converted to a 10-point scale; each selection was multiplied by 2.5 for questions 7 and 8, and by 2 for the other questions, and each score was then summed up based on the highest score of 100.

The survey category consisted of surveys about previous or current medical information experience as well as the experience of using FirstER as a novel way of viewing medical information. First, we asked the reason for requiring medical information from hospitals, which medical information was needed, thoughts on obtaining medical information from the hospital with regard to convenience, and the reasons why the participants wanted to obtain their own medical information. Second, we asked how the participant felt about their medical information being saved on the internet, what the most useful and unnecessary functions of the application were, and for comments on more functions for improving FirstER.

#### In-depth Interview

To acquire more information about the subjects, interview questions about the survey's themes and the patient’s medical information experiences in emergencies were asked. The interview questions were semiconstructed.

### Data Analysis

First, we determined the constitution of demographic features and calculated the mean (SD) for the SUS. Further, we conducted linear regression for the SUS according to the smartphone-use score. Data analyses were conducted with R software (version 3.3.1; R Foundation).

For the survey and in-depth interview, we analyzed the frequency, tendency, and keywords of the answers to each question to determine and categorize the participants’ thoughts on themes.

## Results

### Demographics and Smartphone-Use Score

In total, 1000 participants, which included patients and carers, were recruited in this study. The demographic information of these participants is summarized in Table 1. The study participants included people of different age groups, genders, and marital statuses, and differed in the relationship of the main carer, the area of residence, education level, and status as a patient or carer.

There were more female participants (618/1000, 61.8%) than male participants (382/1000, 38.2%). Furthermore, the average age was 41.4 (SD 13.3) years. Of the 1000 participants, there were 414 (41.4%) patients and 586 (58.6%) carers. The number of participants who self-reported as not married was 35.9% (359/1000), and 64.1% (641/1000) self-reported as married. The main carer of the patients was mostly a spouse or family member (962/1000, 96.2%). Of the 1000 participants, 722 (72.2%) had obtained an education higher than college graduation, and 702 (70.2%) lived in Seoul or the capital area. The mean smartphone-use score was 81.4 (SD 11.2; Table 1).

**Table 1:** Demographics and smartphone-use scores of study participants (n=1000).

Characteristics		Value, n (%)
**Gender**		
	Male	382 (38.2)
	Female	618 (61.8)
**Age in years**		
	19-39	481 (48.1)
	40-59	419 (41.9)
	60+	100 (10.0)
**Marital status**		
	Not married	359 (35.9)
	Married	641 (64.1)
**Patient/carer**		
	Patient	414 (41.4)
	Carer	586 (58.6)
**Respondent's main carer**		
	Spouse/family	962 (96.2)
	Other	38 (3.8)
**Education**		
	College or higher-level education	722 (72.2)
	High school or lower-level education	278 (27.8)
**Residential district**		
	Seoul/capital area	702 (70.2)
	Other	298 (29.8)
**Smartphone-use score**		
	≤60	45 (4.5)
	61-80	356 (35.6)
	≥81	599 (59.9)

### Survey

#### Experience of Obtaining Medical Information With a Legacy System

With respect to requesting medical information from the hospitals they had visited, 34.3% (358/1043) of participants reported having no experience requesting their personal medical information, and 56% (584/1043) reported that the reason for requesting their personal medical information was for their employment company or insurance company. The participants needed various kinds of information about their previous conditions while visiting hospitals and wanted to possess their own medical information to use whenever they wanted (eg, to share it with related people; Table 2).

**Table 2:** Experience of obtaining medical information with a legacy system (n=1000).

Survey question and response options		Value, n (%)
**1. Reasons for requiring medical information from hospitals^a^**		
	No experience	358 (34.3)
	For submission to insurance companies	513(49.2)
	For submission to company or school	71 (6.8)
	For personal storage	17 (1.6)
	Deliver to family or carers	50 (4.8)
	Other	34 (3.3)
**2. How convenient did you feel the process was for requesting medical information before?**		
	Inconvenient	395 (39.5)
	Mediocre	395 (39.5)
	Convenient	142 (14.2)
	No response	68 (6.8)
**3. Have the absence of any of the following kinds of medical information caused you inconvenience when visiting a hospital?^a^**		
	Diagnosis and test results	526 (33.7)
	Medication information in progress (name, capacity, etc)	455 (29.2)
	Types of tests carried out	416 (26.7)
	The name of the hospital visited, or the department of medicine	71 (4.6)
	Other	91 (5.8)
**4. What are your reasons for wanting to get personal medical information?^a^**		
	I want to know all the information related to my health.	685 (28.8)
	I want to have it at all times in case of an emergency.	561 (23.6)
	I think it's my obvious right.	346 (14.5)
	I want to show it to another hospital or another doctor.	295 (12.4)
	I want to show it to my family and my carer.	250 (10.5)
	I want to know my health condition in detail through an internet search or community, etc.	222 (9.3)
	I don't want to collect more detailed medical information than I have now.	16 (0.7)
	Other	6 (0.2)

^a^The respondent could select multiple responses.

#### Experience of Using FirstER

Regarding FirstER, the most helpful information in the application, as per the participants, was ranked in the following order: lab test results (327/1091, 30.0%), personal health self-record (280/1091, 25.7%), function showing data to medical staff (211/1091, 19.3%), and drug information (124/1091, 11.4%). Further, most participants (329/1018, 32.3%) responded that all the information showed on the application was necessary. Personal medical information is sensitive, but many participants (790/1000, 79.0%) agreed to store their medical information on the internet with their consent, while others (207/1000, 20.7%) felt uncomfortable. Table 3 shows participant comments on the application.

**Table 3:** Participant experience of using FirstER (n=1000).

Question and response options		Value, n (%)
**1. The most helpful information in the application^a^**		
	Lab test results	327 (30.0)
	Personal health self-records	280 (25.6)
	Function showing data to medical staff	211 (19.3)
	Medication information	124 (11.4)
	Real-time emergency department records	99 (9.1)
	Hospital visits record	29 (2.7)
	Other	21(1.9)
**2. The most unnecessary information in the application^a^**		
	None	329 (32.3)
	Hospital visits record	111 (10.9)
	Personal health self-records	109 (10.7)
	Real-time emergency department records	79 (7.8)
	Function showing data to medical staff	51 (5.0)
	Lab test results	29 (2.8)
	Medication information	23 (2.3)
	Other	287 (28.2)
**3. Opinion about the acceptability/unacceptability of personal medical information being stored on the internet when consent is given**		
	It does not matter	450 (45.0)
	Not good or bad	340 (34.0)
	Uncomfortable	207 (20.7)
	No response	3 (0.3)
**4. Information that would be useful if additionally provided (a subjective answer)**		
	Expanding nonemergency services	22
	Imaging test results	15
	Improvement in understanding of medical information	15

^a^The respondent could select multiple responses.

### In-depth Interview

Through interviews, we wanted to compare the experience of obtaining medical information with a legacy system with that of using FirstER to see whether FirstER alleviated discomfort.

#### Experience of Obtaining Medical Information With a Legacy System

Interviews showed that participants were keen to know more about their medical information and to participate in their medical care. However, they were not well informed of their medical information, and they were passive in the use of medical information and their own medical care. The following is a description of the difficulties that patients were experiencing concerning their medical information: (1) difficulties getting accurate medical information in a timely manner; (2) difficulties obtaining medical information; (3) diverse reasons for desiring personal medical information.

#### Difficulties Getting Accurate Medical Information in a Timely Manner

The interviewees could not provide exact information when they were asked questions by medical staff because they could not remember, or it was difficult to arrange, medical information in an urgent situation.

There were many things I couldn't think of, and it was difficult to prepare documents about medical information while rushing to the emergency department.

study participant (patient)

#### Difficulty Obtaining Medical Information

In the present system, patients usually obtained their medical information from the hospital. This was often experienced as stressful, as it was time-consuming and many medical information documents or CDs must be prepared.

It took me a while to register a video, and I always had to go to the hospital 30 minutes and an hour early.

study participant (patient)

#### Desire to Obtain Medical Information

The reasons for wanting to obtain one’s own medical information were diverse, and the results obtained were the same as those in the survey: participants wanted to use medical information directly in an emergency, and they needed exact information; also, participants wanted to concurrently look for a second opinion, considering its importance in cases of long-term disease.

I always want to obtain information on my physical condition.

study participant (patient)

#### Experience of Using FirstER

**Figure 4:**
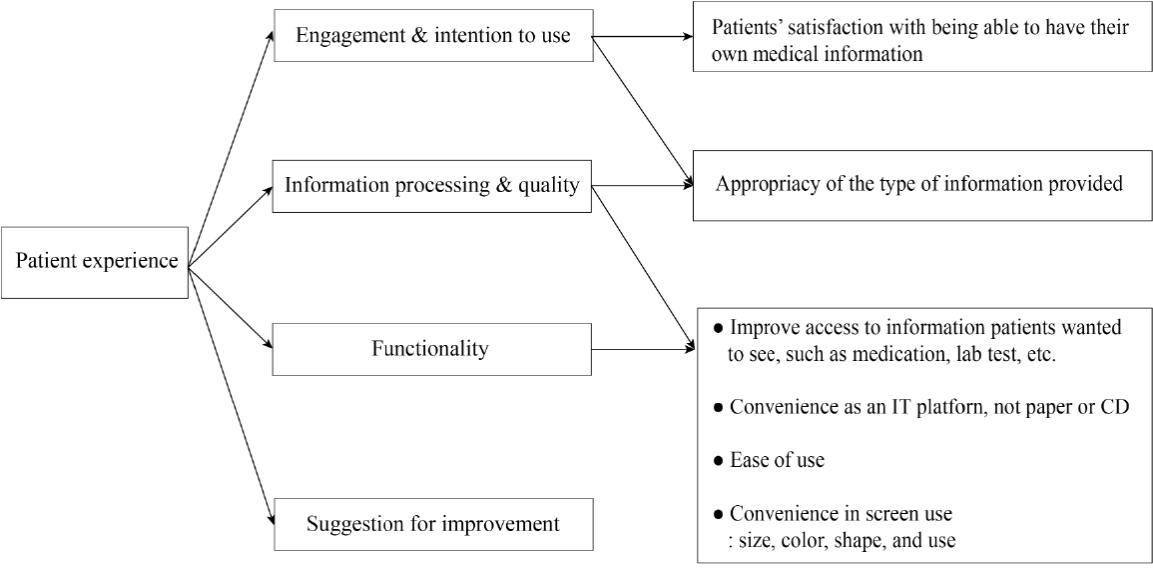
The construction of questions about the user experience of FirstER.

We conducted interviews about FirstER to identify whether patients were satisfied with their experiences with it. We constructed interview questions with 4 categories: engagement, information processing and quality, functionality, and suggestions for improvement [19,23,27,28]. Each category related and interacted with others. Most participants were satisfied with the 4 categories of experience (Figure 4).

#### Engagement and Intention of Use

Questions about engagement examined whether FirstER had satisfied the users' needs for medical information so that they could continue using the application.

It's important that I have my medical information because I've been sick for a long time.

study participant (patient)

I think I'll use it a lot because I can get a lot of information and it is convenient.

study participant (patient)

#### Information Processing and Quality

We asked whether the patients had been provided with the information they wanted and whether the type of information or its contents were sufficient. Most users answered in the affirmative to these questions. Concretely, the participants reported that lab test results and medication information were the most useful. It meant that the participants used FirstER for viewing the medical information they needed. Some participants (7/20, 35.0%) reported that informational variety was lacking in the application, and some (4/20, 20.0%) reported that it was appropriate. This, once again, confirmed that user demand for information is high.

I'm satisfied with the basic medical treatment and prescription medicine.

study participant (carer)

I don’t think we have much information yet. The more, the better.

study participant (patient)

#### Functionality

We asked if FirstER was convenient to use; provided increased access to necessary information; could help with communication with medical staff; and had an appropriate layout, screen color scheme, size features, and navigation. It seemed to be helpful in emergencies, and participants answered that it was more convenient to use an IT platform than to pack various documents. In addition, most of the respondents said that the application was easy to use and was properly configured, but there were opinions that it would be nice to have a screen-magnifying function for the elderly and more explanations about medical terms or figures.

I think it's best that I can show my medical information to the emergency department when I'm in a hurry.

study participant (patient)

Just by several clicks, I can see my blood test result. It was more convenient than using the internet or seeing a document.

study participant (patient)

I think it’s good for the elderly too, because the font is big. Also, it's simple to navigate, so I think it'll be easy for the elderly as well as young people.

study participant (patient)

#### Suggestions for Improvement

Through the users’ opinions for improvement, we wanted to gain insight into the factors that encourage users to continue using the application and to understand the kind of experience patients currently have. Responses to the service were positive. Various opinions were provided regarding improvements, such as a service extension for cooperative hospitals or outpatients and wards, simplifying the authentication and login process, providing additional information like imaging test results, and providing explanations of terminology and information.

I hope many hospitals participate so that I can see lots of other medical information.

study participant (carer)

General people don't know medical terminologies. It would be better with a brief explanation.

study participant (carer)

Multimedia Appendix 1 contains more information about the in-depth interviews.

### SUS Analysis

We evaluated participant usability of FirstER with the System Usability Scale (SUS). The mean SUS score value was 67.1 (SD 13.8), which means participants evaluated FirstER as very near to acceptable [25]. When determining the relationship between smartphone-use scores and SUS values, the correlation coefficient was 0.8887, and it had a significant linear regression (*P*=.0015).

### Summary of User Experience of the Legacy System and FirstER

A summary of the user experience with the legacy system and with FirtER is presented in Table 4.

**Table 4:** Summary of the user experience with the legacy system and with FirstER.

Key theme	Legacy system	FirstER
Engagement and intention to use	Patients were not well aware of their health conditions and medical information, and they were passive in the use of their medical information and treatment. Patients wanted their medical information for proof and for several other reasons, such as sharing with others, preparing for emergencies, etc.	Patients were satisfied with owning their medical information. FirstER improved patient communication with medical staff about their condition.
Information processing and quality	Patients wanted their previous medical information, such as information on lab tests, medication, hospital visit records (hospital and department names), etc.	FirstER provided the information patients had wanted, and the patients found the content to be overall sufficient. Some participants reported that it would have been better if there had been explanations for better understanding of the medical terms.
Functionality	Difficulty obtaining accurate medical information promptly The hassle of getting medical information; time-consuming, many documents, etc.	FirstER enabled better and easier access to medical information using patients ‘mobile phones.

## Discussion

### Principal Results and Strengths of the Study

To the best of our knowledge, this was the first study to introduce mPHR to end-users such as patients and carers in real emergencies that were not covered by conventional PHR studies. It aimed to ensure that mPHRs improve patient access to information and improve patient experience in the emergency department. It is also meaningful to implement the essence of PHR in that it allows patients to collect their own medical records and seek continuous medical care by solving issues of high security, interoperability, and accuracy in patient record delivery with 3 large tertiary hospitals in Korea.

Studies have shown that patients were not well aware of their medical information and were passive in utilizing medical information and medical care. However, patients and carers wanted to own and better utilize medical information and were willing to be active in their medical activities. This study was the first PHR demonstration attempted in emergencies. The purpose was to observe the user experience of FirstER in emergencies; therefore, it is difficult to conclude whether FirstER outperformed the legacy system. However, FirstER fulfilled these patient needs by increasing access to information they previously needed and made it possible to sufficiently use this information, which showed that the introduction of mPHR is reasonable in real emergencies.

### Suitability of Information in FirstER: Improving Patient Experience by Providing Information That Patients Need

Emergency departments function in almost all hospitals in one space, so they require faster and more accurate information and access to as much information as possible [3]. By providing patients with their necessary information, patient experience can be enhanced [5,23]. However, there is no standard for what should be put into the emergency PHR. FirstER contained name, address, birth date, contact information, communication, allergy, and medical history related details [29]. Additionally, it also contained lab test results, medication-related information, etc, that cause information gaps in communication in emergency situations [4].

Medical staff selected the type of information reflected in FirstER, based on prior research on which information points are necessary for patients and medical staff in emergencies [24]. Imaging information was not included in FirstER because of system problems and general patient inability to read them independently. The survey results showed that 32.3% (329/1018) of participants reported no unnecessary information in FirstER, indicating that FirstER contained enough core data needed in emergency situations.

### Reflections on the Low SUS Outcome

After receiving positive responses to FirstER in the survey and interview, the SUS was also expected to be above-average; however, it was not. The reason may be that SUS, which only deals with the ease of use of the application itself, does not contain the experience of medical information retrieval covered in surveys and interviews. Moreover, an unstable environment such as the emergency department may have made it more difficult to use FirstER rather than a typical application.

This demonstration was a pilot study to see the usability of mPHR in emergencies rather than to perfect the application. Usually, to make a better application, it takes a longer time period, and the application must go through several iterative processes. If we can further develop the application based on the results from this study, we can expect a better SUS.  

### Challenges in Application

First, interoperability is critical. In this study, we organized hospitals to participate and share data. However, for extension and everyday use of the service, detailed and extensive discussion is needed, as complexity increases exponentially with the increase in the number of participants. Even within an institution, multiple departments such as outpatient and operating rooms often lack sufficient standardization on patient data, which would result in challenges in a real-world application.

Second, data disclosure and privacy issues are critical. We designed this study using tight security protocols. Based on privacy legislation, the medical cloud zone, an service infrastructure (IaaS) provided by the Samsung Data System (Seoul, Korea), met the facilities and equipment standards necessary for the management and preservation of electronic medical records (EMRs), which is a requirement for the remote storage of EMRs. As for data transfer to each patient, we obtained strict written consent approved by the IRB and other consent forms in the application informing patients of which information we would collect from them. For registration, verification through the patient’s email address was required, and a unique patient’s hospital ID was used as a key to access the patient’s information from the system [30]. In addition, we provided insurance in case of accidents like patient information leakage.

Regarding data disclosure and privacy for real implementation, we have to maintain confidentiality based on regulations. Intensive user verification such as biometric technologies can be used to this end. In addition, there should be a fundamental consensus regarding the range of information sharable with these systems. Providers and users need to actively discuss the types, amounts, and methods of data sharing. In addition, we are required to consider the range of subjects that patients may want to share their data with.

### Limitation

First, this study was conducted in only 3 hospital emergency departments; therefore, there could be a bias in the selection of subjects, and the results may not reflect patients and carers in all emergencies. In addition, because we had interviewed some participants via a convenience sampling method, it was difficult to reflect the opinions of all patients in the emergency department.

Second, this study was conducted within a short period of time, and within that period, visits to the emergency departments were often one-time; therefore, we could not check the participants using FirstER for a longer period of time.

Third, in relation to the above, we have not seen a better medical outcome among the patients, such as medication compliance, self-management, etc. It would have been better to show the practical utility of FirstER if we had identified better medical outcomes than those who did not use it.

### Conclusion

FirstER showed that mPHRs can potentially contribute to enhancing patient experience by providing patients and carers with conveniently accessible medical information in real emergencies. To the best of our knowledge, this is the first study to introduce mPHR to end-users such as patients and carers in real emergencies that were not covered by conventional PHR studies. It is also meaningful to implement the essence of PHR in that it allows patients to manage their own medical records and seek continuous medical care while solving the issues of high security, interoperability, and accuracy.

## Appendix

Multimedia Appendix 1 In-depth interview.

## Abbreviations

AMC: Asan Medical Center
DMC: Dong-A Medical Center
EMR: electronic medical record
IRB: institutional review board
mPHR: mobile personal health record
PHR: personal health record
SMC: Samsung Medical Center
