# Supplementary material for: User Experience of Mobile Personal Health Records for the Emergency Department: Mixed Methods Study
Source: JMIR Mhealth Uhealth. 2020 Dec 15;8(12):e24326. doi: 10.2196/24326 (PMC7772069; doi:10.2196/24326)
Supplement: Multimedia Appendix 1 [file mhealth_v8i12e24326_app1.docx]

[Appendix 1] In-depth Interview

1. Experience of obtaining medical information with a legacy system

- **Have you ever been asked questions about your medical information when you visited hospitals, and could you easily provide exact information when requested?**

“Yes, I could just say my approximate disease name, but couldn’t answer questions about others. ” (carer)

“Yes, it was okay. But I can’t memorize data of my parents all the time because we live in different places, so we can't always watch over them.” (carer)

“ There were many things I couldn't think of, and it was difficult to prepare documents about medical information while rushing to the emergency department.” (patient)

- **Reason for inconvenience in getting medical information**

“I had to call the hospital I went to before to check.” (patient)

“I don't know the exact names of the diseases and the tests I received. …When I rush to the hospital, I don't have CDs containing imaging test results or anything like that. ” (patient)

“It took a while, so I always had to go 30 minutes and an hour early for getting the imaging test results.” (patient)

- **Have you used the app the hospital provides and if you did, which function did you use most?**

“It's almost just a reservation, and then test results.” (patient)

“I'm looking for a blood test or something.” (carer)

- **The most preferred medium for viewing medical information**

“Since I always carry my smartphone with me, I can check it right away. So I think the application will be the most convenient. ”(carer)

“ The most comfortable thing is cell phones, as you might lose paper. However, the older people may be comfortable with paper. Generations like us may be comfortable with applications and cell phones, because we carry around our cell phones. For PCs and things like that, you have to use it in a fixed and designated location, so there are some time and space constraints.” (carer)

- **How much is the desire to obtaining medical information on a 10-point scale and the reason**

“10 points. I always want to obtain information based on my physical condition.” (patient)

“ About 9 points. When I'm in an emergency, and I'm asked this and that, it's kind of annoying.” (patient)

1. **Experience of using FirstER**

- **Which function did you feel was the most useful in FirstER?**

“Well, health records, blood pressure control.” (carer)

“The medication, the first thing I got, the prescription…”(patient)

“The list of medical treatments.” (carer)

- **Is the quantity of information FirstER provides adequate?**

“I thought I was a little short on information.” (carer)

“I think the amount of information provided is adequate.” (patient)

- **Were you satisfied with the size, colors, fonts of letters? Also, was it easy to use and understand the FirstER?**

“The font size, I didn't feel pressured, but I think it would be nice to have the ability to increase the font size, because the elderly are not used to small letters.” (carer)

“All right, it was appropriate. It wasn't hard.” (carer)

“I think it's appropriate. I like the size of the letters. But the elders might think there were a lot of items and find it somewhat complex. ” (carer)

- **Do you think FirstER is helpful and are you willing to use it?**

“Yeah, if it's secure.” (patient)

“I think I'll use it a lot because I can get a lot of information and it's convenient.” (patient)

- **Do you have any idea for improving FirstER?**

“I hope many hospitals participate so that I can see lots of other medical information .” (carer)

“Regarding the lab test results, the general person doesn't know if it's good or bad. I think it would be good to provide a description of that and a reference to the results.” (carer)

“I think it would be good to provide a simple login.” (carer)

“I think it is a little bit less frequent for general people to go to the emergency department even though of course there are many patients who go to the emergency department. Usually there are more people who go to the hospital for outpatient treatment. So, it would be good if we can see the records for outpatients.” (patient)
